# Epilepsy and Autism Spectrum Disorder: An Epidemiological Study in Shanghai, China

**DOI:** 10.3389/fpsyt.2019.00658

**Published:** 2019-09-12

**Authors:** Anyi Zhang, Jijun Li, Yiwen Zhang, Xingming Jin, Jun Ma

**Affiliations:** ^1^Department of Developmental and Behavioral Pediatrics, Shanghai Children’s Medical Center, Shanghai Jiao Tong University School of Medicine, Shanghai, China; ^2^MOE-Shanghai Key Laboratory of Children’s Environmental Health, Shanghai Jiao Tong University School of Medicine, Shanghai, China; ^3^Shanghai Institute of Pediatric Translational Medicine, Shanghai Children’s Medical Center, Shanghai Jiao Tong University School of Medicine, Shanghai, China; ^4^Department of Integrative Medicine on Pediatrics, Shanghai Children’s Medical Center, Shanghai Jiao Tong University School of Medicine, Shanghai, China

**Keywords:** autism spectrum disorder, age, epilepsy, gender, social function neural network

## Abstract

Autism spectrum disorder (ASD) is a neurodevelopmental disease that may involve various brain abnormalities. However, there are few large epidemiological studies on the relation between epilepsy and ASD in terms of different genders and ages. This study aimed to evaluate the relation between epilepsy and ASD based on 74,251 Chinese children aged 3–12 years who were recruited from kindergartens and primary schools in China. ASD was diagnosed according to the Diagnostic and Statistical Manual of Mental Disorders—Fifth Edition (DSM-V), and verification of epilepsy was based on medical records. The enrolled children diagnosed with ASD were examined by magnetic resonance imaging (MRI) and took genetic tests to rule out other neurological and congenital diseases. The raw odds ratio (OR) was 60.53 [95% confidence interval (CI) = 37.80–96.92, P < 0.01] for epilepsy and ASD, and the adjusted OR was 38.99 (95% CI = 20.70–73.41, P < 0.01) after controlling for the confounders. Moreover, the adjusted OR was significantly higher in girls (OR = 45.26, 95% CI = 16.42–124.76, P < 0.01) than in boys (OR = 32.64, 95% CI = 14.33–74.34, P < 0.01). Among children with younger age, the adjusted OR was the highest (OR = 75.12, 95% CI = 22.80–247.48.16, P < 0.01). These findings suggest that epilepsy might be closely linked to the development of ASD, especially for early-onset epilepsy and among girls.

## Introduction

Autism spectrum disorders (ASDs) have been increasingly prevalent in recent years, with an estimated incidence changing from 1 in every 110 children to 1 in 88 children (1, 2). Large epidemiologic studies have shown that males are two to three times more likely to suffer from ASD than females (3–5). It is characterized by impaired social interaction and communication, repeated and stereotyped behaviors, and restricted interest in activities, in accordance with the Diagnostic and Statistical Manual of Mental Disorders—Fifth Edition (DSM-V) (6). As a heterogeneous neurodevelopmental disorder, it can also bring a great burden to patients and their families (7). There are many genetic and environmental factors for ASD identified in previous studies. A family history of psychiatric diseases, especially in first-degree relatives or siblings, is one of the most important risk factors. Other factors include advanced paternal age, gestational influence, maternal unemployment, etc. (8–12). Previous studies reported that ASD had co-occurred with several developmental and psychiatric disorders, including intellectual disability (ID), language delay, attention-deficit hyperactivity disorder, tourette’s syndrome, anxiety, and depression (11). Moreover, the disorder of neurodevelopment was considered as the main pathogenesis of ASD. Children with ASD had impaired social brain networks and disrupted functional connectivity in attention networks (13, 14). Studies focusing on the pathophysiology of ASD also reported that early onset of abnormal neurogenesis, neurite growth, and signal pathways were also involved in developing ASD (15).

Epilepsy in childhood is a prevalent neurologic disease that affects patients’ social interaction, communication skills, and patterns of behavior (16). Previous studies on the risk factors of epilepsy have shown that cranial trauma, infections in the central nervous system, difficult labor, and family history of epilepsy were associated with childhood epilepsy (17). In addition, epilepsy is more common in ASD children, with a rate varying from 8% to 30% (18–20). There is also a strong influence on the patients’ quality of life and well-being in epilepsy and ASD in childhood. Despite some studies focusing on the association between ASD and epilepsy (21–24), most of them had a small study sample; only a few were population-based studies (20, 25). They described a high rate of ASD and epilepsy without confounders. However, they did not consider the impact of onset age of epilepsy on ASD prevalence, either. Additionally, there were no proper criteria on diagnosis of ASD in some previous reports.

In summary, our study aims to investigate the association between ASD and epilepsy after adjusting for the potential confounding factors, including age, sex, advanced paternal age, social economic factors, etc. Furthermore, sensitivity analysis is applied to figure out whether the onset age of epilepsy and gender have an influence on the association.

## Materials and Methods

### Subjects and Study Design

This was a population-based cross-sectional study conducted in China in June 2014, and it was designed as an important section of the governmental population survey about ASD (26). According to the geographical and social population distribution in Shanghai, there were 17 districts, including 8 in the central districts and the remaining 9 in the suburban districts, in the study. Then the participants were recruited from seven randomly chosen districts (three urban districts: Yangpu, Xuhui, Jing’an, and four suburban districts: Minhang, Pudong, Fengxian and Chongming) using a random number generator. In each district, kindergartens and primary schools were chosen randomly from the list of schools. In total, 134 kindergartens and 70 primary schools were included. Moreover, considering that children attending special education schools were at higher risk of ASD, we enrolled all children in special education schools in these seven districts, since in China, children with mental retardation, language impairment, or hearing impairment are recommended to attend special education school. Additionally, another seven special education schools were included too. Altogether, 84,075 children in general school and 859 children in special school were sampled in our survey.

We trained the teachers to distribute the questionnaires to the recruited students, students were asked to bring the questionnaires home, and parents were requested to fill in the questionnaires. All participants and their caregivers signed the informed consent, and they were also informed that participation would be innominate and voluntary. The Human Research Ethics Committee of Shanghai Children’s Medical Center, Shanghai Jiao Tong University School of Medicine, had approved the study protocol and ethnical application. Meanwhile, this survey was approved by the Institutional Review Board of Shanghai Municipal Commission of Health and Family Planning.

**Figure 1:**
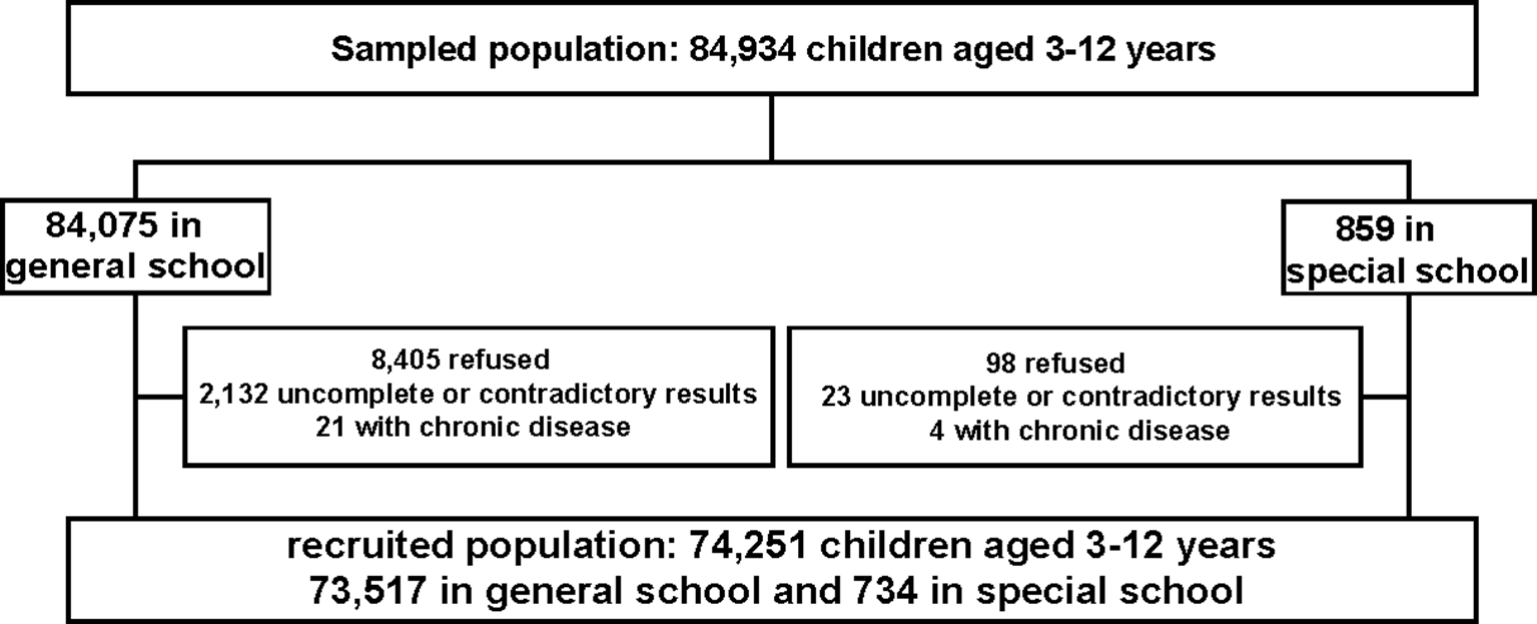
Detailed participation diagram.

The exclusion criteria: firstly, questionnaires with contradictory results between parents and teachers were excluded from our study; secondly, questionnaires with more than 67% questions uncompleted were considered as invalid (27); thirdly, children with chronic diseases such as chronic kidney disorders and tumors were excluded from our research. Finally, the participants were 74,251 schoolchildren in total, with a mean age of 7.59 ± 2.32 years old (734 in special school and 73,517 in general school). The diagram of the study is shown in **Figure 1**.

### Questionnaires

There were four questionnaires in our study: children’s family social environment and growth questionnaire, children’s Social Communication Questionnaire (SCQ) (Chinese version) (28), children’s behavior and mood development questionnaire, and obesity-related health condition questionnaire.

In the first questionnaire, parents or caregivers were asked to offer information involving their children’s fundamental condition (gender, height, weight, birth weight, health condition, TV time, etc.); the mother’s pregnancy (age of pregnancy, physical and mental health); and the family social environment (character of parents or other caregivers, their educational background, family income). The second questionnaire described the participants’ social communication; we used the Chinese version of the SCQ. It included 40 questions regarding language development, repeated and stereotypical behaviors, and restricted interest in activities. Questions related to children’s attention and mood were asked in questionnaire 3. Questions regarding life style associated with obesity were asked in the last questionnaire, such as the frequency of eating snacks, eating breakfast, and doing exercise, and food allergy. More detailed information about all questionnaires is shown in the **Supplementary Material**. In this study, we used only two questionnaires out of four; the others were described in other publications.

### Diagnostic Criteria

ASD was defined according to the *DSM-V*, with deficits in social communication and interaction in multiple situations, and restricted and repeated behavior and interests as the core features (11). Other features included language developmental delay, motor delay and abnormalities, and other atypical developmental abnormalities. The second questionnaire, the Chinese version of the SCQ, was widely used as a screening tool for ASD children whose chronological ages were beyond 4 years old, or mental ages beyond 2 years old (29). The SCQ contained 40 yes/no questions answered by parents. A score ≥15 was considered to be positive, and then the teacher would be asked to completed the SCQ. If the result was still positive, the child was recommended to visit a physician who specialized in developmental and behavioral pediatrics at Shanghai Children’s Medical Center, and these children took further cognitive tests and behavior evaluations. At least two experienced pediatricians made a diagnosis according to the *DSM-V*. Once diagnosed with ASD, the patient was examined by a comprehensive examination including gene tests and magnetic resonance imaging (MRI) to exclude inherited metabolic disorders and neurostructural disorders.

Epilepsy was diagnosed based on the *International Classification of Diseases 10^th^ version*, as unproved epileptic seizure with abnormal electroencephalogram (EEG), and was divided into two subgroups according to the involved brain regions (29). In our questionnaire, parents were asked about the diagnoses of epilepsy made by a neurological pediatrician for those who chose epilepsy as a co-morbidity.

### Statistical Analysis

We used total number and prevalence to describe categorical data, and mean and standard deviation for numerical data. Comparison of the categorical data was performed using χ^2^ tests, and two-sample t-tests were used to compare the numerical data. After that, we performed logistic regression to evaluate the association between ASD and epilepsy. First, univariate analysis was conducted to determine the potential risk factors. The selected variables were then involved in multivariate logistic regression, controlling for age, sex, and body mass index (BMI). Moreover, the impact of the onset age of epilepsy on ASD was assessed by multivariable logistic regression. Results were recorded using odds ratios (ORs) and 95% confidence intervals (CIs). We performed the analysis in Statistical Program for Social Science (SPSS) software (version 19.0), and significance was set at P <0.05 (two-tailed).

## Results

### Baseline Information

We collected the information of 74,251 schoolchildren in total in our study, with a mean (SD) age of 7.59 (2.32) years old, 39,034 (53.3%) boys and 34,264 (46.7%) girls. When compared to normal children, those with ASD had an older age. ASD was more prevalent in boys [146 (0.4%)] than girls [46 (0.1%)]. In these subjects, the prevalence of diagnosed ASD was 192 in 74,251 (0.26%), and in children with ASD, the prevalence of epilepsy was much higher [22 (11.5%)], while the total prevalence of epilepsy was 0.2% (180/74,251) (**Table 1**). Moreover, the prevalence of epilepsy in ASD was even higher in special education schools (14.4%), since all cases of ASD and epilepsy co-occurrence were found in the special education schools (**Supplementary Table 1**). The performance of ASD children in school was not as good as normal children. Additionally, advanced paternal and maternal age also existed in children with ASD. Moreover, 153 ASD children were found in the special education schools; see **Supplementary Table 1** in the **Supplementary Material**. Of 192 children with ASD, 73 were examined by MRI, 133 took a genetic test, and eventually, all of them were included in the study.

**Table 1:** Comparison of subjects’ sociodemographic characteristics.

Characteristics	Total (N = 74,251)	ASD (N = 192)	Normal (N = 74,059)	P value^a^
Age, mean (SD)	7.59 (2.32)	8.20 (2.44)	7.59 (2.32)	<0.01
Male, n (%)	39,034 (53.3%)	146 (0.4%)	38,888 (99.6%)	<0.01
Female n (%)	34,264 (46.7%)	46 (0.1%)	34,218 (99.9%)	
BMI, mean (SD)	16.98(3.68)	17.96 (3.99)	16.97 (3.67)	<0.01
Epilepsy, n (%)	180 (0.2%)	22 (11.5%)	158 (0.2%)	<0.01
School record				
Excellent	31,733 (42.8%)	12 (6.3%)	31,721 (42.9%)	<0.01
Good	33,673 (45.4%)	54 (28.4%)	33,619 (45.4%)	
Average	5,483 (7.4%)	46 (24.2%)	5,437 (7.3%)	
Poor	1,506 (2.0%)	71 (37.4%)	1,435 (1.9%)	
Single parent	3,171 (4.4%)	9(4.8%)	3,162 (4.3%)	0.77
Only child	52,386 (71.1%)	137 (71.7%)	52,249(71.1%)	0.84
Advanced maternal age^b^	3,562 (5.0%)	11 (5.8%)	3,551(5.0%)	<0.01
Advanced paternal age^b^	8,774 (12.8%)	30 (17.2%)	8,744 (12.8%)	0.02
Household income^c^				
< 4,625	9,557 (13.2%)	32 (17.0%)	9,525 (13.2%)	0.40
4,625–15,420	27,640 (38.2%)	65 (34.6%)	27,575 (38.2%)	
15,420–46,259	23,238 (32.1%)	58 (30.9%)	23,180 (32.1%)	
> 46,259	12,008 (16.6%)	33 (17.6%)	11,975 (16.6%)	

ASD, autism spectrum disorder; SD, standard deviation; BMI, body mass index. ^a^Unpaired t-tests were adopted to compare the differences of the data, and chi-square tests for categorical data; ^b^advanced maternal and paternal age means 35 years old or older; ^c^the measurement of household income is in US dollars per year.

### Risk Factors for ASD

**Table 2** shows the results of the risk factors for ASD determined by univariable logistic regression. According to the raw ORs, it is suggested that male gender, older age, higher BMI value, epilepsy, advanced paternal age, higher parents’ education level, gestational factors including depression and nervousness during pregnancy, parents’ unsociable characteristic, and overindulging in caregiving could increase the risk of ASD, in which epilepsy was significantly associated with ASD, accompanied by a raw OR (95% CI) of up to 60.53 (37.80–96.92).

**Table 2:** The potential risk factors for ASD by univariable logistical regression.

Variables	No.	ORs	95% CIs	P value
Male gender	39,034 (53.3%)	2.8 4	2.03 –3.96	<0.01
Age	–	1.01	1.00–1.01	<0.01
BMI	–	1.05	1.03–1.09	<0.01
Only child	52,386 (71.1%)	1.03	0.75–1.42	0.84
Epilepsy	180 (0.2%)	60.53	37.80–96.92	<0.01
Advanced age^a^				
Paternal	8,774 (12.8%)	1.42	0.96–2.10	0.08
Maternal	3,562 (5.0%)	1.18	0.64–2.17	0.59
Pregnancy psychology				
Depression	2,411 (3.2%)	3.44	2.11–5.63	<0.01
Nervous	3,449 (4.6%)	3.07	1.98–4.78	<0.01
High education level^b^				
Father	22,771 (30.7%)	2.02	1.52–2.69	<0.01
Mother	19,967 (26.9%)	1.60	1.19–2.14	<0.01
Unsociable father	10,676 (14.4%)	2.33	1.69–3.02	<0.01
Unsociable mother	6,253 (8.4%)	1.81	1.20–2.72	<0.01
Low household income^c^	9,557 (13.2%)	1.35	0.92–1.98	0.12
Overindulgence	7,981 (10.7%)	2.18	1.52–3.12	<0.01

No, number of people; ORs, odds ratios; 95% CIs, 95% confidence intervals. ^a^Advanced age means 35 years old or older; ^b^high education level means having a bachelor’s, master’s, or doctor’ s degree; ^c^Less than 4,625 dollars per year is considered low household income. - Age and BMI are numerical data and cannot be coded into number and percent.

### Association Between ASD and Epilepsy

**Table 3** provides the results of multivariable logistic regression, showing the association between epilepsy and ASD after adjusting for the potential risk factors decided by univariable logistic regression. Overall, there was strong evidence to suggest that children with epilepsy were at an increased risk of co-occurring ASD (OR = 38.99, 95% CI = 20.70–73.41, P < 0.01). In addition, our data also revealed a statistically significant association between ASD and the following variables: male gender, older age, higher BMI value, gestational factors including depression and nervousness during pregnancy, higher paternal education level, paternal unsociable characteristic, and overindulging in caregiving, while advanced paternal reproductive age, higher maternal education level, and lower household income were confounding factors in our study.

**Table 3:** The association between ASD and epilepsy using multivariable logistical regression test.

Variables	No.	ORs	95% CIs	P value
Epilepsy	180 (0.2%)	38.99	20.70–73.41	<0.01
Male gender	39,034 (53.3%)	2.76	1.85–4.13	<0.01
Age	–	1.01	1.01–1.02	<0.01
BMI	–	1.04	1.00–1.08	0.08
Advanced paternal age^a^	8,774 (12.8%)	1.43	0.91–2.25	0.12
Pregnancy psychology				
Depression	2,411 (3.2%)	3.05	1.66–5.61	<0.01
Nervous	3,449 (4.6%)	2.59	1.48–4.53	<0.01
High education level^b^				
Father	22,771 (30.7%)	1.36	1.10–1.68	<0.01
Mother	19,967 (26.9%)	1.13	0.92–1.39	0.24
Unsociable father	10,676 (14.4%)	1.72	1.14–2.59	0.01
Unsociable mother	6,253 (8.4%)	1.28	0.75–2.18	0.37
Overindulgence	7,981 (10.7%)	1.81	1.19–2.77	<0.01

^a^Advanced age means 35 years old or older; ^b^high education level means having a bachelor’s, master’s, or doctor’ s degree. - Age and BMI are numerical data and cannot be coded into number and percent.

### Sensitivity Analysis

**Figure 2:**
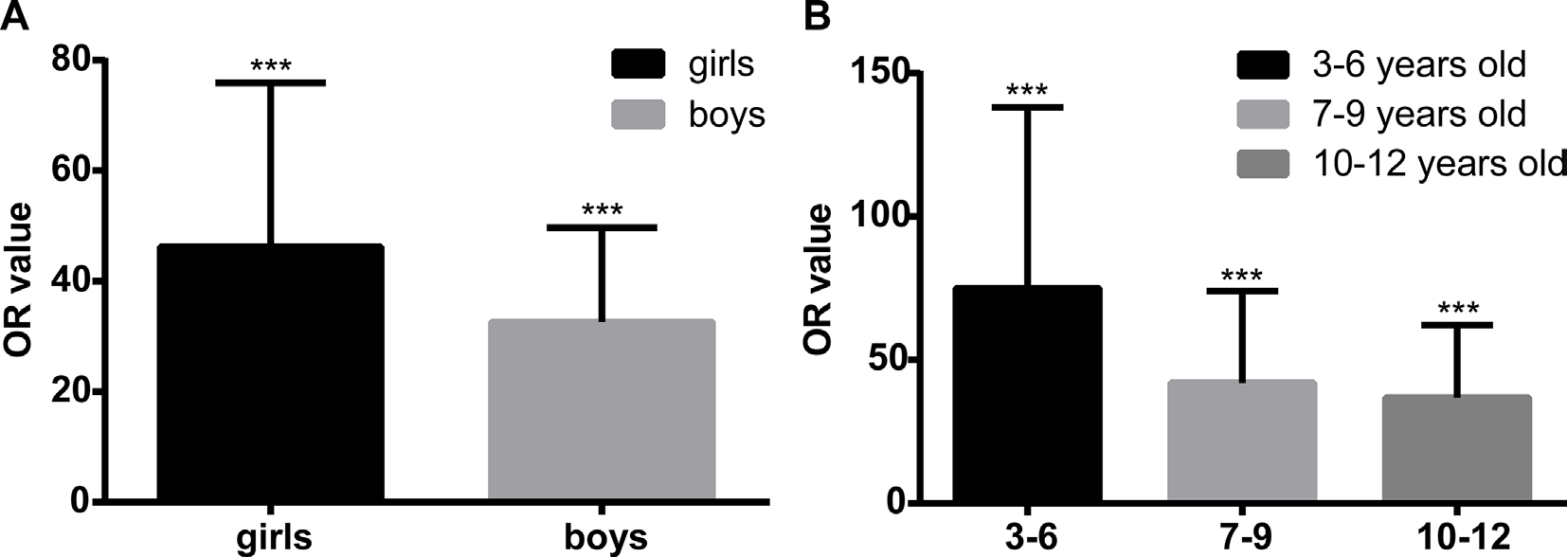
Sensitivity analysis of the association between epilepsy and autism spectrum disorder (ASD) in different genders and age groups. Panel **(A)** shows the multivariable logistic regression in different genders. After adjusting for the co-variables, including age, body mass index (BMI), paternal reproductive age, parents’ education level, gestational factors including depression and nervousness during pregnancy, parents’ unsociable characteristic, and overindulging in caregiving, the effect size of the association between epilepsy and ASD is higher in girls [odds ratio (OR) = 45.26, 95% confidence interval (CI) = 16.42–124.76, P < 0.01] than in boys (OR = 32.64, 95% CI = 14.33–74.34, P < 0.01). Panel **(B)** shows the multivariable logistic regression in different age groups. After adjusting for the co-variables, including age, BMI, paternal reproductive age, parents’ education level, gestational factors including depression and nervousness during pregnancy, parents’ unsociable characteristic, and overindulging in caregiving, the effect size of the association between epilepsy and ASD is at the highest level in the youngest group (OR = 75.12, 95% CI = 22.80–247.48, P < 0.01), and in age groups 2 and 3, the OR values are 42.09 (95% CI = 13.93–127.22, P < 0.01) and 36.98 (95% CI = 13.33–102.58, P < 0.01), respectively. ***P < 0.001.

We performed extended multivariable logistic regression models to evaluate whether age and gender might have an influence on the association between epilepsy and ASD. The association seemed more evident in girls than in boys, as the ORs (95% CIs) were 45.26 (16.42–124.76, P < 0.01) and 32.64 (14.33–74.34, P < 0.01), respectively. We divided all participants into three subgroups according to their age: 1) 3–6 years old (31,729, 43.1%); 2) 7–9 years old (27,593, 37.2%); and 3) 10–12 years old (14,224, 19.2%). We can see from **Figure 2** that after adjusting for the co-variables mentioned in **Table 3**, the association between epilepsy and ASD was the strongest in the youngest subgroup (OR = 75.12, 95% CI = 22.80–247.48, P < 0.01), and the OR decreased with age; in age group 2, it was 42.09 (95% CI = 13.93–127.22, P < 0.01), and 36.98 (95% CI = 13.33–102.58, P < 0.01) in children who were 10–12 years old.

## Discussion

The findings of this study including 74,251 schoolchildren suggest that the overall risk for epilepsy associated with ASD is robust, and the excess risk observed in girls and younger children is substantial. Moreover, this study has an appropriate sample size and includes potential risk factors, especially in the different ages and genders, to assess the association of epilepsy and ASD in Chinese children

This study also found that epilepsy was positively associated with ASD, which was highlighted in girls and early onset of epilepsy. Taken together with the previous research assessing the association between epilepsy and ASD (18–20, 30), this study indicates an urgent need for further studies to explore the underlying mechanism. We summarized the possibilities to explain the pathogenesis for the association. Firstly, previous studies have suggested that neurobiological dysfunction was believed to be important in ASD. Previous studies have also suggested the abnormal acceleration of brain growth in early childhood with ASD (15), and disorders in consistent networks of brain regions, especially the social network, were closely linked with ASD (31–33). As highlighted in an increasing number of functional MRI (fMRI) articles, the atypical connectivity of brain networks in ASD included the superior temporal gyri (34), inferior frontal gyri (34), insula (35), default mode network (36), cingulate region (37), and parieto-occipital region (38). Meanwhile, the recurrent unprovoked seizures might also be harmful to the brain regions, especially when they occur at the regions mentioned above. These reasons might contribute in the same way to the development of ASD. Additionally, the early onset of epilepsy might cause damage to brain regions and therefore pose an increased risk for co-occurring ASD. Moreover, seizures could cause structural damage in the developing brain (39); the structural abnormality of the social brain network would lead to ASD (40). Recurrent epilepsy in the long term reflects underlying brain dysfunction (39), making the central nervous system more vulnerable. When the social network regions are damaged, it could lead to the development of ASD or make ASD worse.

Secondly, epilepsy and ASD might share a common pathophysiology. There is accumulating evidence of ASD causative genes involving up to 1,000 genes (41), and genetics also plays an important role in epilepsy (42). The genes affecting the developmental process of neuron migration, cell–cell adhesion, neurite growth, synapse formation, synaptogenesis, translation, intracellular transport, synaptic plasticity, and synaptic function, including astrotactin 2 (ASTN2), autism susceptibility candidate 2 (AUTS2), contactin-associated protein-like 2 (CNTNAP2), postsynaptic density protein-95 (DLG4), fragile X mental retardation 1 (FMR1), gephyrin (GPHN), lissencephaly 1 (LIS1), methyl CpG binding protein 2 (MECP2), neurexin 1/2/3 (NRXN1/2/3), phosphatase and tensin homolog (PTEN), reelin (RELN), synapsin 1/2/3 (SYN1/2/3), T-Box brain protein 1 (TBR1), tuberous sclerosis 2 (TSC2), and ubiquitin protein ligase E3A (UBE3A), were reported to be associated with both ASD and epilepsy (15). In ASD, the abnormality in both serotonin and γ-aminobutyric-acid (GABA) systems, due to the alteration of brain serotonin synthesis capacity, and reduction in the expression of GABA synthetic enzymes and receptors were demonstrated consistently; ASD children had an abnormal social activities (11). In epilepsy, decreased GABA receptors in the central nervous system were reported to be important in the epileptic occurrence (43).

Thirdly, gestational factors such as complications and infection during pregnancy and neonatal disorders might also affect the association between ASD and epilepsy positively (11, 44, 45). It is reported that these gestational factors might affect neurodevelopment and have a long-term influence on children during their life span. Meanwhile, environmental factors including exposure to chemicals during pregnancy might positively influence the association between ASD and epilepsy, too (11, 46).

It is worth mentioning that the co-occurrence of epilepsy and ASD was higher in children attending the special education schools. Nearly all children with ID have been enrolled in special education schools in Shanghai, where ID prevalence is quite high. It was reported that ID is a generalized brain disorder that was particularly common in children with epilepsy since it could damage the neurons; the prevalence of epilepsy was much higher in ID children than their normal peers (47). Meanwhile, there was also a high rate of concurrent ASD in ID children (48). Hence, it is not difficult to understand why the co-morbidity of epilepsy with ASD was much higher in children attending the special education schools.

As confirmed in our study of Chinese children, sex, age, paternal education level, paternal unsociable characteristics, and overindulging in caregiving are consistent risk factors. A previous study reported that ASD affected almost 4 to 5 times more boys than girls, and in our study, boys are 2.79 times more likely to have ASD (11, 49). Advanced paternal age is another risk factor for ASD, although the underlying pathogenesis is still unclear, which is supposed to be associated with germline mutation, especially in fathers (50, 51). However, in our study, the association between ASD and advanced paternal reproductive age was not significant. In addition, the prevalence of ASD in children with higher paternal education level is 1.36 times more than those with lower paternal education, and this is consistent with the previous studies reporting that the parents of ASD children might be good at engineering/math training or be more technically talented than are other parents (11, 52, 53). A unsociable parent might have a poor parent–infant interaction, which might influence the infant’s social environment in early childhood, but this theory needs further research (54). The underlying mechanism of the association between ASD and overindulging in caregiving is still unknown.

The main strengths of this study were the large sample size, the adjustment for potential confounding and risk factors, and the use of a validated instrument for ASD and epilepsy diagnosis. However, there was no follow-up for this epidemiological study; there might exist a recall bias, as the information was gathered based on the parents’ memory. Thirdly, there might exist some bias in the enrollment, as parents of children with severe problems may not have been willing to participate in this study.

In conclusion, epilepsy is significantly associated with ASD after controlling for the confounders, and the association is stronger among girls and children with an early onset of epilepsy.

## Ethics Statement

All participants and their caregivers had signed the informed consent, and they were also informed that the whole survey involvement would be innominate and voluntary. The Human Research Ethics Committee of Shanghai Children’s Medical Center, Shanghai Jiao Tong University School of Medicine had approved the study protocol and ethnical application. Meanwhile, this survey was approved by the Institutional Review Board of Shanghai Municipal commission of Health and Family Planning.

## Author Contributions

AZ and JL co-designed the study, conducted the analysis, and drafted the initial manuscript. YZ and XJ contributed to the conduct of the study. JM designed the study and reviewed and revised the manuscript. All authors reviewed the manuscript and approved the final version.

## Funding

This study was funded by grants from the Science and Technology Commission of Shanghai Municipality (grants 14JC1404600, 16411952800, and 18411960200), the Development of Science and Technology in Pu Dong District (PKJ2017-Y06), and the National Natural Science Foundation of China (81528023).

## Conflict of Interest Statement

The authors declare that the research was conducted in the absence of any commercial or financial relationships that could be construed as a potential conflict of interest.
