# Supplementary material for: Epilepsy and Autism Spectrum Disorder: An Epidemiological Study in Shanghai, China
Source: Front Psychiatry. 2019 Sep 12;10:658. doi: 10.3389/fpsyt.2019.00658 (PMC6751887; doi:10.3389/fpsyt.2019.00658)
Supplement: Supplementary file 1 [file DataSheet_1.docx]

Epilepsy and Autism Spectrum Disorder: An Epidemiological Study in Shanghai, China

Anyi Zhang^1,2,3#^, Jijun Li^4#^, Yiwen Zhang^1,2,3^, Xingming Jin^1,2,3^ Jun Ma^1,2,3*^

**Running title**: Epilepsy and autism spectrum disorder

**Author affiliations:** 1.Department of Developmental and Behavioral Pediatrics, Shanghai Children’s Medical Center, Shanghai Jiao Tong University School of Medicine, Shanghai, 200127, China; 2. MOE-Shanghai Key Laboratory of children’s Environmental Health, Shanghai Jiao Tong University School of Medicine, Shanghai, 200127, China; 3. Shanghai Institute of Pediatric Translational Medicine, Shanghai Children’s Medical Center, Shanghai Jiao Tong University School of Medicine, Shanghai, 200127, China; 4. Department of Integrative Medicine on Pediatrics, Shanghai Children’s Medical Center, Shanghai Jiao Tong University School of Medicine, Shanghai, 200127, China

#The two authors contribute equally to this work.

***Corresponding author**: Jun Ma, Department of Developmental and Behavioral Pediatrics, Shanghai Children’s Medical Center, 1678 Dong fang Road, Shanghai, China, 200127 (E-mail: [majun@shsmu.edu.cn](mailto:majun@shsmu.edu.cn)).

**Supplementary material**

**Questionnaires**

There are four questionnaires in our study.

No.1 The baseline information and social economic factors. There are 44 questions, 1. The gender of the children: boy or girl; 2. The hometown of the children: local or not; 3. The birthdate of the children; 4. The birth weight of the children; 5. Gestational age: early-term, full-term, late-term; 6. The way of giving birth: normal birth or caesarean; 7. The only child or not; 8. Whether the mother having a history of abortion before; 9. The condition of the child right after birth; 10. Feeding pattern: breast feeding, mixed feeding or artificial feeding; 11. Being diagnosed of other diseases: neonatal jaundice, brain trauma, febrile seizure, epilepsy, enuresis, others; 12. Whether having sever systemic diseases; 13. The height and weight of the child; 14. Whether going through traumatic events or not; 15. The time that the child spend on playing online games per day: <1 hour, 1-2 hours, 2-3 hours, 3-4 hours, 4-5 hours, >5 hours; 16. The time that the child spend on watching TV on weekday per day: <1 hour, 1-2 hours, 2-3 hours, >3 hours; 17. The time that the child spend on watching TV on weekend per day: <1 hour, 1-2 hours, 2-3 hours, >3 hours; 18. The frequency of eating between-meal nibbles per day: never, once, twice, three times, more than three times; 19. The frequency of eating snack before sleep per week: never, once, twice, three times, more than three times; 20. The mother’s age when pregnant; 21. The father’s age when pregnant; 22. The mother’s height and weight; 23. The father’s height and weight; 24. Assisted reproductive techniques used or not; 25. The mother’s emotion when pregnant: happy, depressive, and nervous; 26. When did the depressive mood appear firstly: never, early pregnancy, middle pregnancy, late pregnancy; 27. How long did the depressive mood last: never, less than one week, one week to one month, one month to three months; three months to a half year; more than a half year; 28. The mother’s health condition when pregnant; 29. Family history of psychiatric disease; 30. Single parent family or not; 31. The parents’ relationship; 32. The character of father: open or unsociable; 33. The character of mother: open or unsociable; 34. The education level of father: illiterate, primary school, middle school, high school/vocational school, college, university, postgraduate or above; 35. The education level of mother: illiterate, primary school, middle school, high school/vocational school, college, university, postgraduate or above; 36. The education level of the person who does the most job in raising the child: illiterate, primary school, middle school, high school/vocational school, college, university, postgraduate or above; 37. Who does the most job in raising the child: the parent, the grandparent, the babysitter; 38. Does the mother smoke or not; 39. Does the father smoke or not; 40. Does the father drink or not; 41. Does the mother drink or not; 42. The health condition of the person who does the most job in raising the child: good or poor; 43. The parenting pattern: overindulgence, violence, and nothing special; 44. The family annual income: less than 10,000 RMB, 10,000-30,000 RMB, 30,000-50,000 RMB, 50,000-100,000 RMB, 100,000-150,000 RMB, 150,000-200,000 RMB, 200,000-1,000,000 RMB, more than 1,000,000 RMB.

No. 2 The Chinese version of SCQ. SCQ is widely used as a screening scale for ASD in children whose chronological ages are beyond 4 years old, or mental ages beyond 2 years old (Barnard-Bark et al, 2016). The SCQ contained 40 yes/no questions regarding the language development, repeated and stereotypical behaviors and restricted interests activities.

No. 3 The children's behavioral and emotional development questionnaire. No. 4 The life style associated with obesity in early childhood. The questions in questionnaire No.3 and No.4 are not included in our study.

Supplement Table 1. Comparison of ASD patients’ sociodemographic characteristics.

| characteristics | Total  (N=192) | General school  (N=39) | Special education school  (N=153) | P value^a^ |
| --- | --- | --- | --- | --- |
| Age mean (SD) | 8.20 (2.44) | 5.92 (2.62) | 8.79 (2.01) | <0.01 |
| Male n (%) | 146 (0.4%) | 32 (82.1%) | 114 (74.5%) | 0.35 |
| Female n (%) | 46 (0.1%) | 7 (17.9%) | 39 (25.5%) |  |
| BMI mean (SD) | 17.96 (3.99) | 16.95 (2.70) | 18.22 (4.22) | 0.09 |
| Epilepsy n (%) | 22 (11.5%) | 0 (0.0%) | 22 (14.4%) | <0.01 |
| School record |  |  |  | 0.01 |
| Excellent | 12 (6.3%) | 7 (17.9%) | 5 (3.3%) |  |
| Good | 54 (28.4%) | 12 (30.8%) | 42 (27.8%) |  |
| Average | 46 (24.2%) | 9 (23.1%) | 37 (24.5%) |  |
| Poor | 71 (37.4%) | 10 (25.6%) | 61 (40.4%) |  |
| Single-parent | 9 (4.8%) | 1 (2.6%) | 8 (5.3%) | 0.49 |
| The only child | 137 (71.7%) | 33 (84.6%) | 104 (68.4%) | 0.05 |
| Advanced maternal age^b^ | 11 (5.8%) | 2 (5.3%) | 9 (6.0%) | 0.12 |
| Advanced paternal age^b^ | 30 (17.2%) | 9 (24.3%) | 21 (15.3%) | 0.29 |
| Household income^c^ |  |  |  | 0.79 |
| <4625 | 32 (17.0%) | 4 (10.3%) | 28 (18.8%) |  |
| 4625-15420 | 65 (34.6%) | 12 (30.8%) | 53 (35.6%) |  |
| 15420-46259 | 58 (30.9%) | 14 (35.8%) | 44 (29.5%) |  |
| >46259 | 33 (17.6%) | 9 (23.1%) | 24 (16.1%) |  |

Abbreviations: ASD, autism spectrum disorder; SD, standard deviation; BMI, body mass index.

^a^ unpaired t-tests were adopted to compare the difference of the data, and Chi-square tests for categorical data; ^b^ advanced maternal and paternal age means 35 years old or older;^c^ the measurement of household income is US dollars per year.

**REFERENCES**

Barnard-Bark, L., Brewer, A., Chesnut, S., Richman, D. and Schaeffer, A. M. (2016). The sensitivity and specificity of the social communication questionnaire for autism spectrum with respect to age. Autism Res. 9, 838-845. doi: 10.1002/aur.1584.
